# Comparison of processing approaches for single-cell analysis of esophageal biopsy samples

**DOI:** 10.1016/j.jacig.2026.100757

**Published:** 2026-07-01

**Authors:** Paramita Dutta, Kevin Okamoto, Kira Chaiboonma, Carolyn H. Baloh, Tanya M. Laidlaw, Sourya Bhattacharyya, Ferhat Ay, Seema S. Aceves

**Affiliations:** a La Jolla Institute of Immunology, La Jolla, Calif; b Division of Allergy and Immunology, Department of Pediatrics, University of California, La Jolla, Calif; c Rady Children’s Hospital, San Diego, Calif; d Immune Tolerance Network, Division of Allergy & Clinical Immunology, Brigham and Women's Hospital, Harvard Medical School, Boston, Mass

**Keywords:** Eosinophil, eosinophilic esophagitis, transcriptomics, epithelial cell, fibroblast

## Abstract

**Background:**

Eosinophilic esophagitis is an eosinophil-rich type 2 inflammatory disease with increasing global prevalence. Multicenter randomized trials are critical, but use of certain cutting-edge techniques, such as single-cell sequencing, can be challenging owing to a requisite need for immediate tissue processing.

**Methods:**

We performed a pilot single-cell RNA sequencing (scRNA-seq) study using single esophageal biopsy samples in 3 processing protocols. Biopsy samples were processed as (1) fresh, (2) immediately frozen, or (3) frozen after dispersion to single cells. scRNA-seq was performed using 10× Genomics to understand cell type preservation despite freezing.

**Results:**

Biopsy samples from 4 patients were processed with 1 or more of each of the fresh and frozen protocols. Comparison of the scRNA-seq profiles of frozen samples with those obtained using the criterion standard of fresh biopsy samples demonstrated that epithelial and mast cell transcriptomes were preserved in frozen dispersed cells and frozen tissues. Lymphocytes, myeloid cells, endothelial cells, and fibroblasts were present in all of the fresh samples. Fibroblast, myeloid, and endothelial cells were lost in immediately frozen tissue but relatively preserved in tissue dispersed to single cells and then frozen. All 9 epithelial cell clusters present in the fresh samples were preserved in dispersed frozen cells but variably retrieved from directly frozen tissue.

**Conclusion:**

Single esophageal biopsy specimens can be used successfully in scRNA-Seq when samples are dispersed and frozen as single cells. Myeloid cell, endothelial cell, and fibroblast transcriptomes, as well as nondifferentiated epithelial cell transcriptomes, are not preserved when tissue samples are frozen without initial dispersion to single cells.

## Introduction

Eosinophilic esophagitis (EoE) is a type 2 food antigen–mediated chronic disease. Progressive EoE leads to pathologic tissue remodeling with esophageal rigidity and lost luminal diameter.^1^^,^^2^ Single-cell RNA sequencing (scRNA-seq) is increasingly being used to profile the gene expression of EoE structural and immune cells.^3^^,^^4^ Multicenter trials are being performed in eosinophilic gastrointestinal diseases (EGIDs) and oral immunotherapy (OIT) antigen-tolerizing protocols, which may induce an EGID.^5^, ^6^, ^7^, ^8^, ^9^ However, these clinical studies are limited in their ability to utilize techniques such as scRNA-seq because tissue storage and shipment between multiple sites can reduce cell viability. The differences between esophageal cell transcriptomes that are derived using fresh versus frozen conditions and the optimal fixation or freezing protocols for small pieces of esophageal tissue have yet to be systematically analyzed for compatibility with scRNA-seq. To better understand the potential use of scRNA-seq following freezing and tissue storage, we aimed to characterize the retention of esophageal and immune cell transcriptomes from esophageal biopsy samples dispersed immediately to single cells and then frozen (frozen cells) or from directly frozen tissue that was later processed to single cells (frozen tissue) as compared with the criterion standard of immediately processed of biopsy samples (fresh samples).

For detailed methods, see the Supplementary Methods in the Online Repository (available at www.jaci-global.org).

## Results and discussion

**Fig 1:**
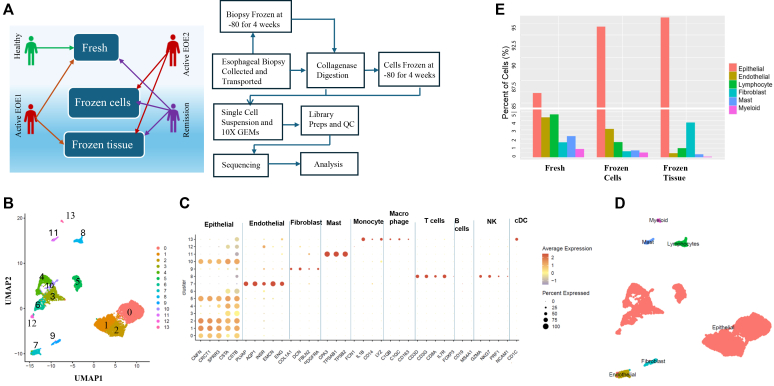
Study overview and cell type annotation. **A,** Overview of the study protocol. **B,** Uniform Manifold Approximation and Projection (UMAP) visualization and clusters for the complete data set across all of the biopsy samples. **C,** Dot plot of gene expression corresponding to the output clusters with respect to marker genes of different cell types. **D,** Annotated clusters with major cell types according to marker gene expression. **E,** Percentage of cells belonging to individual cell types for sample conditions. *cDC*, Conventional dendritic cell; *GEMS*, Gel Beads-in-Emulsion; NK, natural killer; Preps*,* preparations; *QC,* quality control.

We performed a pilot study using pediatric esophageal biopsy samples in a frozen storage protocol to mimic the storage conditions potentially used in a multicenter trial (Fig 1, *A*). Using single biopsy samples for each processing protocol from 1 healthy control (HC), 2 participants with active EoE (participants EoE-A1 and EoE-A2), and 1 participant with EoE in remission (EoE-R) (Table I), we were able to generate 8 single-cell libraries for the 3 conditions (fresh, frozen tissue, and frozen cells [Table I]). Frozen tissue or cells were processed for scRNA-seq after 4 weeks at –80°C. The EoE-R samples yielded 3 single-cell libraries with adequate cell numbers from each processing condition. The HC sample yielded adequate cell numbers from fresh processing. The biopsy samples from participants EoE-A1 and EoE-A2 generated adequate number of cells for scRNA-seq in 2 of the 3 conditions; fresh and frozen tissue for participant EoE-A1 and frozen cells and frozen tissue for participant EoE-A2 (Fig 1 and Table I). Analyses of cell numbers and genes detected per cell showed variation across processing conditions and tissue samples, which was as expected when working with limited clinical specimens (Table I).

**Table I:** Demographic, histologic, and transcriptomic data on participants

Processing	Participant and disease state	Cell count	Mean reads/cell	Median transcripts/cell	Sex/age	Peak eosinophils per hpf (D/M/P)	Location used for scRNA-Seq	BZH//DIS/LPF
Fresh	Control	1,665	144,573	630	F/16 y	0/**0**/0	Middle	No/no/no LP
	EoE remission	7,189	78,294	570	M/11 y	9/**0**/0	Middle	No/no/no LP
	EoE active-A1	826	119,656	1,620	F/12 y	34/33/17	Distal	Yes/yes/yes
Frozen cells	EoE active-A2	2,907	88,036	124	F/10 y	3**/2**/35	Middle	No/no/no LP
	EoE remission	3,637	80,594	1,079	M/11 y	9/0/0	Middle	No/no/no LP
Frozen tissue	EoE active-A2	1,703	244,208	644	F/10 y	3/2/35	Middle	No/no/no LP
	EoE active-A1	446	603,066	369	F/12 y	34/33/17	Distal	Yes/yes/yes
	EoE remission	1,174	279,990	904	M/11 y	9/0/0	Middle	No/no/no LP

*BZH*, Basal zone hyperplasia; *D*, distal; *DIS*, dilated intercellular spaces; *LPF*, lamina propria fibrosis; *M,* middle*; P*, proximal.

### Across-patient analyses

To characterize scRNA-seq data from the different conditions, we harmonized the data across all 8 single-cell libraries (see the Supplementary Methods) and obtained 14 clusters (Fig 1, *B*). Using literature-derived marker genes for esophageal tissue,^4^ we annotated the major cell types as “structural” (epithelial, endothelial, and fibroblast) or “immune” (mast cells, monocytes, macrophages, T cells, B cells, natural killer cells, and dendritic cells). Eosinophil transcriptomes were not detected (Fig 1, *C* and *D*). There was substantial representation of immune cells in the fresh biopsy samples, which was not the case for the frozen samples (Fig 1, *E*). In accordance with this, the fraction of epithelial cells was higher in the frozen cells and frozen tissue than in the fresh samples. To ensure that our data matched published data sets of dysregulated genes in the EoE-A participants versus in the EoE-R participant versus in the HC, we utilized a curated gene set. As expected, levels of *CCL26*/eotaxin-3 and *ALOX15* were increased in the EoE-A participants versus in the EoE-R participant and in both versus in the HC (see Fig E1 in the Online Repository at www.jaci-global.org). We used epithelial-specific marker genes^4^ to identify epithelial subtypes as quiescent, proliferating, transitioning 1, transitioning 2, differentiation low, and differentiation high (see Fig E2 in the Online Repository at www.jaci-global.org).

We found the presence of transcriptomes that define T cells (*CD3E*, *CD3D*, and *CD8A*), natural killer cells (*GZMA*, *NKG7*, and *PRF1*), monocytes/macrophages (*CD14*, *CD163*), and conventional dendritic cells (*CD1c*) (Fig 1, *C*). B cells (*CD19* or CD20/*MS4A1*) were difficult to detect within the lymphocyte cluster. Mast cell transcripts were readily detected (*CPA3* and *TPSB1/B2*) (Fig 1, *C*).

### Within-patient analyses

**Fig 2:**
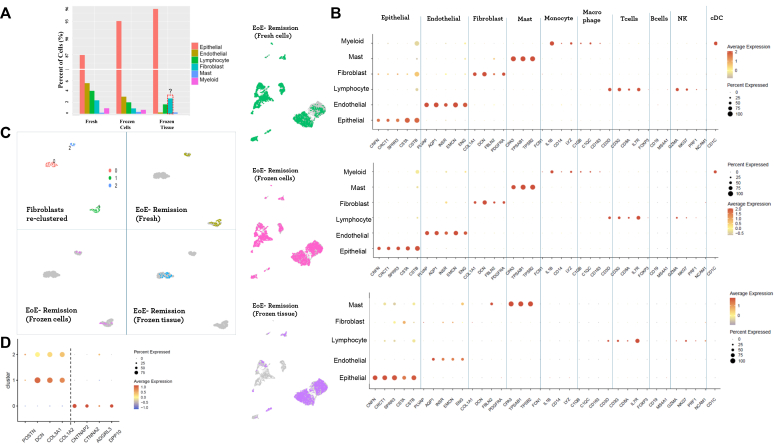
Characterization of a patient with EoE in remission for different sample conditions: **A,** Percentage of cells belonging to individual cell types for different samples. Question mark indicates cells presumed to be fibroblasts. **B,** Uniform Manifold Approximation and Projection (UMAPs) and dot plots of gene expression of marker genes in fresh, frozen cells, and frozen tissue. **C,** UMAPs of subclustered fibroblasts; cells are highlighted by processing protocol. **D,** Dot plot of gene expression for marker genes of fibroblast and glial cells for the subclusters obtained in (**C**).

Because disease status could be a confounding factor affecting the observed differences in preserved cell types by processing, we used the EoE-R participant samples to directly compare the differences between the effect of processing conditions on single biopsy samples from the same esophageal level from the same patient. This is important because transcriptomes and the prevalence of fibroblasts, immune cells, and certain epithelial cell subsets are variable by disease state and activity. We found that lymphocyte transcriptomes decreased in frozen versus in fresh processing protocols and were the least abundant in frozen tissue (Fig 2, *A* and *B*). Mast cell transcriptomes were well preserved through both freezing protocols (Fig 2). Myeloid cell transcriptomes were relatively preserved in frozen cells but undetectable in frozen tissue (Fig 2).

On initial analysis, it appeared that epithelial cell transcriptomes were preserved in both freezing protocols (Figs 1, *E* and 2, *A* and *B*). However, when epithelial cells were analyzed by subtype, 6 different epithelial cell subtypes (9 distinct clusters) were present in the fresh biopsy samples (see Fig E3, *A* and *B*). Although all of these clusters and subtypes were preserved in frozen cells, mainly differentiated (high and low) epithelial cells (clusters 0, 1, and 2) were preserved in frozen tissue (see Fig E2, *B* and *C*). Endothelial cells were relatively preserved in frozen cells but diminished in frozen tissue (Fig 2, *A* and *B*).

Our initial analysis suggested that like epithelial cells, fibroblasts were well preserved in all 3 processing conditions (Fig 2, *A*). However, gene annotation demonstrated an absence of expression for canonical fibroblast markers (eg, *COL1A1, DCN*) in cells from frozen tissue (Fig 2, *B* [*bottom panel*]). This finding prompted us to further evaluate this specific cluster in detail. Additional subclustering of cluster 9 (Fig 1, *B* and *D*) highlighted the fact that that only 2 of 3 subclusters expressed fibroblast marker genes (clusters 1 and 2 in Fig *2, C*). Cluster 0 expressed none of the canonical fibroblast genes but did express glial cell genes (*CNTNAP2, CTNNA2, ADGRL3,* and *DPP10*) and was present only in frozen tissue (Fig 2, *D*). Glial cell transcripts were previously reported in scRNA-seq data sets from patients with EoE.^3^ Together, these data support the idea that esophageal immune and structural cells are best preserved if tissue is dispersed to frozen cells before scRNA-seq. In addition, our analysis suggests that a deep dive into those genes demarcating cell types is required when defining cell subtypes, especially for the epithelial and fibroblast cell compartments; fibroblasts constitute only a very small portion of the overall biopsy sample.

Herein, we have documented our experience piloting a protocol for scRNA-seq that could be used in multicenter studies requiring freezing and transport of esophageal biopsy specimens. We found that processing tissue to create a single-cell suspension before freezing better preserves immune and architectural cell transcriptomes than does whole tissue freezing followed by dispersion. Like our study, scRNA-seq studies done using nasal polyps processed as fresh, frozen cells, or frozen tissue demonstrated that mast cells were preserved by using dispersed frozen cell preparations.^10^ However, in nasal polyps, mast cell transcriptomes were not retained in frozen tissue. These data suggest that tissue type, and, perhaps, biopsy size, influences mast cell survival. Further, we were able to recapitulate the known transcriptomes of immune-associated EoE epithelium and fibroblasts. The strength of our study is that it supports the use of a single biopsy sample dispersed to single cells and frozen as a protocol for future multicenter EGID trials or OIT. These types of investigations would augment the depth of our knowledge.

Our study has limitations. Our sample size was small, and although we aimed to obtain 3 scRNA-seq data sets from each participant, insufficient cell and transcriptome yield limited our ability to do so. Future studies following this cell dispersion and freezing protocol would likely benefit from pooling biopsy samples to achieve higher cell yields. The presence of lamina propria is an inherent challenge when assessing EoE biopsy samples because only 50% of EoE-A samples will contain this deep tissue and EoE-R and HC biopsy samples are even less likely to have lamina propria. In alignment with this, we found that only participant EoE-A1 had lamina propria. In addition, only the biopsy sample from participant EoE-A1 had histologic epithelial abnormalities, including basal zone hyperplasia and dilated intercellular spaces. None of these features were found in any of the other biopsy samples used. EoE, like other EGIDs, is a diffuse but patchy disease, so it is possible that biopsy samples procured from the same esophageal level will differ in their degree of eosinophilia and architectural features. This was particularly salient when assessing the sample from participant EoE-A2, which we coded as active based on the standard EoE histologic diagnostic parameter of at least 15 eosinophils per high power field. However, the level of the biopsy sample utilized was not from the most active esophageal region. We relied on the pathology report from the participant’s medical record to determine histologic disease activity, but it is possible that the sequenced biopsy sample differed from the archived specimen in terms of its histology. Until other scRNA-seq techniques, such as spatial transcriptomics, which allow the alignment between histology and single-cell gene expression, are applied at scale to EoE biopsy samples, this issue is inherent for all scRNA-seq data sets.

Despite these limitations, we were able to recapitulate the expected curated dysregulated genes in participants with EoE versus in the HC. Further, by dispersing cells before cryopreservation, there is a capacity to retain transcriptomes from epithelial clusters as well as from endothelial cells, fibroblasts, T cells, myeloid cells, and mast cells. As such, significant amounts of transcriptomic information could be gleaned from multicenter trials if cell dispersion protocols are optimized and standardized. The decreasing cost and increasing options of scRNA-seq platforms will allow a better understanding of tissue-resident and infiltrating cells during EoE therapeutic trials as well as clinical trials that run a risk of EoE onset, such as food OIT studies. With this knowledge in hand, the intersection between distinctly immunologically mediated food hypersensitivity reactions can be better dissected.

**Clinical implications**

Single esophageal biopsy samples can be used and processed for scRNA-seq following freezing, which could facilitate their use in multicenter clinical trials.

## Disclosure statement

This research was performed as a project of the Immune Tolerance Network, a clinical research consortium headquartered at the Benaroya Research Institute and supported by the 10.13039/100000060National Institute of Allergy and Infectious Diseases of the 10.13039/100000002National Institutes of Health (grant UM1AI109565). The content is solely the responsibility of the authors and does not necessarily represent the official views of the National Institutes of Health. The Aceves laboratory is additionally funded by the Campaign Urging Research for Eosinophilic Disease (CURED) Foundation and the 10.13039/100000062National Institute of Diabetes and Digestive and Kidney Diseases (grant R01DK114457). This study was supported in part by the National Institute of Diabetes and Digestive and Kidney Diseases-funded San Diego Digestive Diseases Research Center (P30DK120515).

Disclosure of potential conflict of interest: S. S. Aceves is a consultant for AstraZeneca, Regeneron, Sanofi, Phathom, FirstTrack, and Ferring Pharmaceuticals; and coinventor of budesonide oral suspension (Eohilia, licensed and produced by Takeda Pharmaceuticals), which is patented by the University of California San Diego. F. Ay serves as a consultant for Allergy & Asthma Medical Group and Research Center. T. M. Laidlaw has received honorarium fees for scientific advisory board consulting from Sanofi-Genzyme, Regeneron, AstraZeneca, and Eli Lilly, as well as research funding from Sanofi-Genzyme. The rest of the authors declare that they have no relevant conflicts of interest.

## Supplementary data

Supplementary Fig E1

Supplementary Fig E2

Supplementary Fig E3
