# Supplementary figures and images for: Comparison of processing approaches for single-cell analysis of esophageal biopsy samples
Source: J Allergy Clin Immunol Glob. 2026 Jul 1;5(5):100757. doi: 10.1016/j.jacig.2026.100757 (PMC13425826; doi:10.1016/j.jacig.2026.100757)

## Slide 1
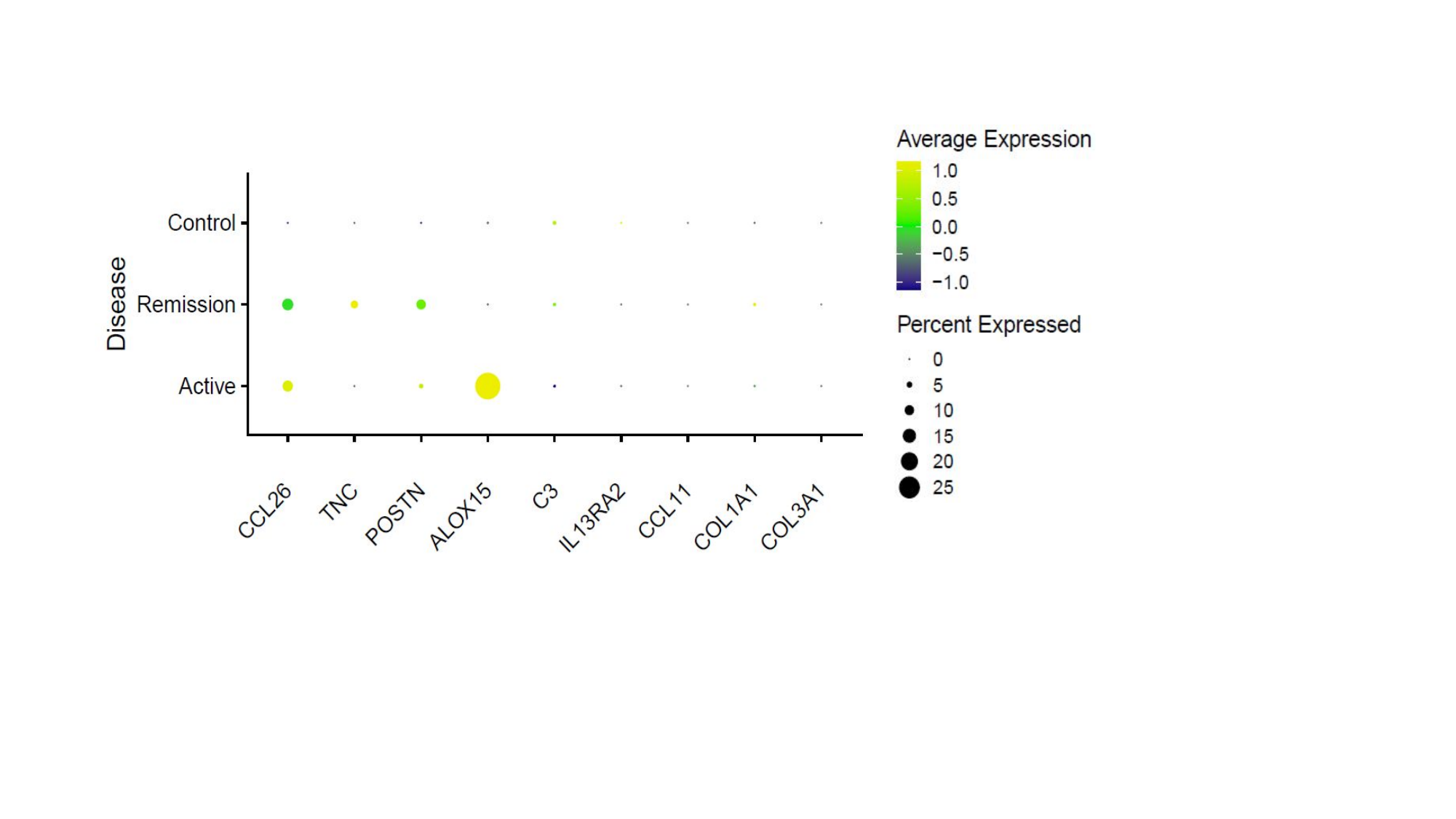

Supplement: Supplementary Fig E1 [file mmc1.pptx]
