# Supplementary material for: Comparison of processing approaches for single-cell analysis of esophageal biopsy samples
Source: J Allergy Clin Immunol Glob. 2026 Jul 1;5(5):100757. doi: 10.1016/j.jacig.2026.100757 (PMC13425826; doi:10.1016/j.jacig.2026.100757)
Supplement: Supplementary Fig E2 [file mmc2.pptx]

## Slide 1
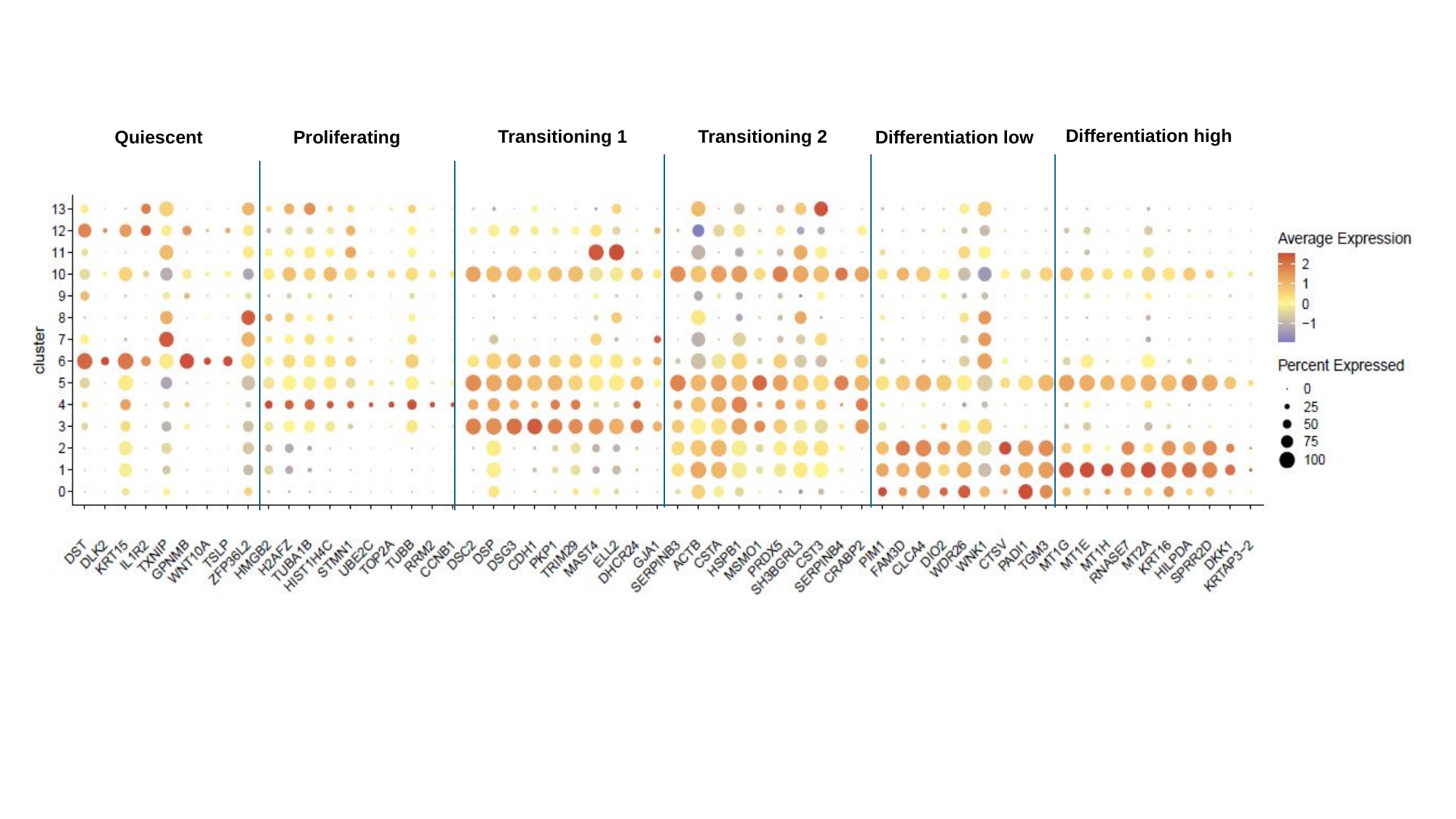

Differentiation high
Transitioning 2
Transitioning 1
Quiescent
Proliferating
Differentiation low
