# Supplementary material for: Comparison of processing approaches for single-cell analysis of esophageal biopsy samples
Source: J Allergy Clin Immunol Glob. 2026 Jul 1;5(5):100757. doi: 10.1016/j.jacig.2026.100757 (PMC13425826; doi:10.1016/j.jacig.2026.100757)
Supplement: Supplementary Fig E3 [file mmc3.pptx]

## Slide 1
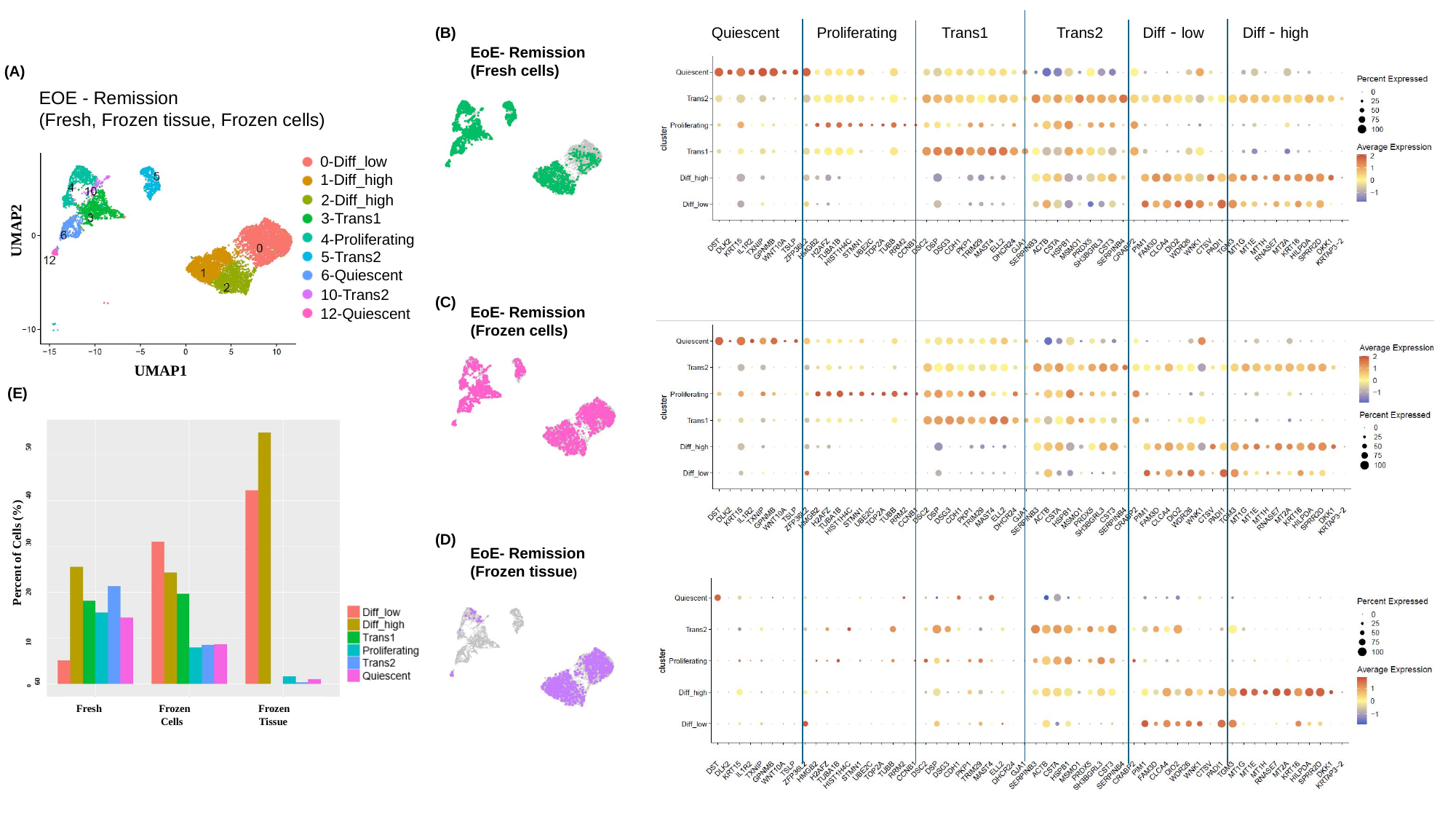

Diff - low
Diff - high
(B)
Quiescent
Proliferating
Trans1
Trans2
EoE- Remission
(Fresh cells)
(A)
EOE - Remission
(Fresh, Frozen tissue, Frozen cells)
0-Diff_low
 1-Diff_high
 2-Diff_high
UMAP2
 3-Trans1
 4-Proliferating
 5-Trans2
 6-Quiescent
10-Trans2
12-Quiescent
UMAP1
(C)
EoE- Remission
(Frozen cells)
(E)
Percent of Cells (%)
0 10 20 30 40 50 60
Fresh Frozen Frozen
 Cells Tissue
(D)
EoE- Remission
(Frozen tissue)
