# Supplementary material for: Comparison of processing approaches for single-cell analysis of esophageal biopsy samples
Source: J Allergy Clin Immunol Glob. 2026 Jul 1;5(5):100757. doi: 10.1016/j.jacig.2026.100757 (PMC13425826; doi:10.1016/j.jacig.2026.100757)
Supplement: Supplementary Data [file mmc4.docx]

**Online Repository**

**Comparison of Processing Approaches for Single Cell Analysis of Esophageal Biopsies**

Paramita Dutta* MSc^1^, Kevin Okamoto* BS^2^, Kira Chaiboonma BS^2,4^, Carolyn H. Baloh MD^3^, Tanya M. Laidlaw MD^3^, Sourya Bhattacharyya PhD**^1^, Ferhat Ay PhD^#1,3^, Seema S. Aceves MD, PhD^#2,4,5^

*These two authors contributed equally

^#^Corresponding author

** Present address: Empirico, San Diego, CA 92130, USA

**Methods** (Online Repository)

**Experimental protocol:**

Samples were collected from patients enrolled in an IRB approved umbrella protocol for studying characteristics and inflammatory markers in children with EoE (UCSD protocol 181690). Samples were collected at Rady Children's Hospital San Diego and immediately transported on 4°C ice pack separated by a thin layer of paper towels in a styrofoam cooler) to UCSD ~ 15-20 min drive.

**Processing single cell RNA-seq data:**

We processed the scRNA-seq fastq.gz files from individual samples using Cell Ranger (version 7.1.0) *count* routine, with respect to the reference genome hg38. Output feature barcoded matrices from Cell Ranger were first applied to decontX (1) and subsequently the decontaminated contact matrices were applied to Scrublet (2) for doublet detection. Then we employed Seurat (3, 4) to process these matrices, and retained cells having nFeature_RNA > 200**,** nFeature_RNA < 7500, percent.mt < 15, percent.ribo < 30 and percent.hb < 0.15. In process, we retained 9,028 cells from all the samples. Then we merged all these samples using the *merge()* routine of Seurat, performed normalization using the method *LogNormalize* with a scaling factor of 10000, obtained top 2000 variable features using the function *FindVariableFeatures*, scaled data (using the routine *ScaleData*()) by regressing with respect to percent.mt, and executed PCA with a dimension of 30. Batch correction was performed by Harmony (5) using the samples as the covariate. Seurat function *FindNeighbors*() with k=20 was used to find the nearest neighbors. Clustering was performed by the Louvain algorithm and using resolution = 0.4. UMAP visualization of Seurat output clusters, gene expression dot plots, and bar plots of cell abundance were generated using Seurat routines.

**Dot plot analysis:**

We visualized the expression levels and percent of cells expressing a gene using dot plot visualization of Seurat with *DotPlot()* function. These genes included marker genes for cell types and cell subsets derived from the literature as well as The Human Protein Atlas (6).

**References**

1. Yang S, Corbett SE, Koga Y, Wang Z, Johnson WE, Yajima M, et al. Decontamination of ambient RNA in single-cell RNA-seq with DecontX. Genome Biol. 2020;21(1):57.

2. Wolock SL, Lopez R, Klein AM. Scrublet: Computational Identification of Cell Doublets in Single-Cell Transcriptomic Data. Cell Syst. 2019;8(4):281-91 e9.

3. Stuart T, Butler A, Hoffman P, Hafemeister C, Papalexi E, Mauck WM, 3rd, et al. Comprehensive Integration of Single-Cell Data. Cell. 2019;177(7):1888-902 e21.

4. Butler A, Hoffman P, Smibert P, Papalexi E, Satija R. Integrating single-cell transcriptomic data across different conditions, technologies, and species. Nat Biotechnol. 2018;36(5):411-20.

5. Korsunsky I, Millard N, Fan J, Slowikowski K, Zhang F, Wei K, et al. Fast, sensitive and accurate integration of single-cell data with Harmony. Nat Methods. 2019;16(12):1289-96.

6. Uhlen M, Fagerberg L, Hallstrom BM, Lindskog C, Oksvold P, Mardinoglu A, et al. Proteomics. Tissue-based map of the human proteome. Science. 2015;347(6220):1260419.

**Supplementary Figure Legends**

**Figure 1. Supplementary Figure 1: Characterizing Fresh biopsy samples across disease states:** Dot plot of curated EoE disease state marker genes for fresh biopsy samples from three patients representing each disease state.

**Figure 2. Supplementary Figure 2: Characterizing subtypes of Epithelial cells across all biopsies:** Dot plot of curated EoE disease state marker genes for fresh biopsy samples from three patients representing each disease state. Dot plot of gene expression of the complete dataset for different marker genes of various Epithelial cell subtypes.

**Figure 3. Characterizing subtypes of Epithelial cells across EoE-Remission samples: (A)** UMAP visualization and annotated clusters for the EoE-R2 sample, annotating subtypes of Epithelial cells. **(B)** Percentage of cells belonging to individual cell types for different sample conditions. **(C)** UMAPs (left) and dot plots of gene expression with respect to various marker genes of different cell types (right) for the Fresh (top), Frozen cells (middle), and Frozen tissue.
